# Splenic Abscess: An Uncommon Entity with Potentially Life-Threatening Evolution

**DOI:** 10.1155/2018/8610657

**Published:** 2018-01-31

**Authors:** Mei-Chun Lee, Chun-Ming Lee

**Affiliations:** ^1^Department of Pharmacy, MacKay Memorial Hospital, Taipei, Taiwan; ^2^Mackay Junior College of Medicine, Nursing and Management, Taipei, Taiwan; ^3^Division of Infectious Disease, Department of Internal Medicine, MacKay Memorial Hospital, Taipei, Taiwan; ^4^Taipei Medical University, Taipei, Taiwan; ^5^MacKay Medical College, New Taipei City, Taiwan; ^6^Department of Internal Medicine, St. Joseph's Hospital, Yunlin County, Taiwan

## Abstract

**Background/Purpose:**

Splenic abscess is rare with potentially life-threatening evolution. The aim of this study is to review the clinical features, microbiological etiologies, treatment, and outcomes of patients with splenic abscess.

**Methods:**

We reviewed the admitted patients with suspected splenic abscess and made the diagnosis of splenic abscess. The clinical characteristics, underlying diseases, treatment course, organism spectra, abscess number and size, therapeutic methods, and clinical outcome at a tertiary medical center in Taiwan over a period of 5 years were analyzed.

**Results:**

Of 16 patients with splenic abscess, the male to female ratio was 1 : 1. Common presentations were fever (11 patients, 68.7%), diffuse abdominal pain (6 patients, 37.5%), left upper quadrant pain or tenderness (6 patients, 37.5%), and left-sided pleural effusions (8 patients, 50%). Antimicrobial therapy was administered in all patients. Fourteen (87.5%) patients recovered under medical treatment. One (6.2%) patient underwent splenectomy, four (25%) patients performed percutaneous drainage of their splenic abscess, and 11 (68.7%) patients received antimicrobial therapy alone.

**Conclusion:**

We noted that mortality may be more related to patients with underlying immunodeficiency. Patients with splenic abscesses receiving antimicrobial therapy alone were in a relatively high proportion and got a good prognosis especially in patients with small and multiple abscesses.

## 1. Introduction

Splenic abscess is an uncommon infection. The incidence of splenic abscess in autopsy studies is estimated to be 0.05–0.7% [1, 2]. Hematogenous spread is the most common cause of splenic abscess. It typically results from endocarditis or seeding from some contiguous sites of infection [3, 4]. Other risk groups include immunosuppressed patients, hemoglobinopathies, and diabetes mellitus [3, 5]. Early diagnosis can readily be made by the combination of computed tomography (CT), abdominal ultrasonography (US), and clinical features [5]. The management of splenic abscesses includes medical therapy, CT-guided percutaneous aspiration, and splenectomy. Recent studies have stressed the changing clinical spectrum and indicated that intravenous antimicrobial therapy alone for patients with splenic abscess showed better outcome [2, 6, 7]. The aim of the study was to review the clinical features, microbiological etiologies, treatment, and outcomes of patients with splenic abscess over the previous 5 years.

## 2. Materials and Methods

### 2.1. Study Design and Data Collection

Admitted patients with the diagnosis of splenic abscess were collected over a period of 5 years (from January 2012 to December 2016). Inclusion criteria of this study were as follows: (1) histological results of the resected splenic tissue showed the presence of an abscess, (2) causative pathogens were isolated from a splenic aspirate or blood culture with compatible imaging studies of CT or US, (3) splenic abscesses were found during exploratory laparotomy, or (4) clinical manifestations were consistent with imaging findings and there was an improvement in the patient's clinical condition after antimicrobial therapy. Age, sex, clinical manifestations, underlying diseases, imaging studies, such as US or CT, treatment course, organism spectra, abscess number and size, therapeutic methods, and clinical outcome were collected and analyzed. The patients were followed up to discharge from our hospital as an end point to define the outcome. This study has been approved by the MacKay Memorial Hospital Institutional Review Board, and the IRB number is 17 MMHIS040.

### 2.2. Statistical Analysis

A univariate analysis of prognostic factors for splenic abscess including age, sex, abscess number, underlying disease, pathogens, and treatment methods was assessed using Fisher's exact test. Continuous variables were compared using the independent *t*-tests such as the mean age and the length of stay. A *p* value of less than 0.05 was considered statistically significant, and the two-tailed test was adopted for all probabilities. All statistical analyses were performed with SPSS version 20.0 (SPSS, Chicago, IL, USA).

## 3. Results

One hundred and four patients with a diagnosis of splenic abscess were reviewed between January 2012 and December 2016, and 16 patients met the criteria for splenic abscess. The male to female ratio was 1 : 1, with a mean age of 49.9 years (range: 1 day to 52 years). Clinical characteristics of the patients including age, gender, predisposing factors, the number of splenic abscesses, microbiological etiologies, treatment methods, and outcomes are shown in Table 1. Of the 16 patients, 5 (31.2%) had diabetes mellitus, 5 (31.2%) had pancreatitis, 4 (25%) had undergone previous abdominal surgery, 3 (18.7%) had liver cirrhosis or chronic liver disease, 3 (18.7%) had contiguous abdominal sites of infection, 2 (12.5%) had HIV infection, 2 (12.5%) had HBV infection, 2 (12.5%) had malignancy, 2 (12.5%) had end-stage kidney disease, and 2 (12.5%) suffered from infective endocarditis.

Causative pathogens were identified in 11 patients (68.7%) with splenic abscess and sterile in the other 5 patients. Blood culture was positive in 7 of 16 patients (43.7%), and abscess culture was positive in 3 of 16 patients (18.7%). Two patients were diagnosed with *Mycobacterium* infection clinically, including patient #14, whose clinical condition improved after treatment with antimycobacterial agents. A systemic candidiasis with spleen invasion was diagnosed in patient #15, in whose blood culture *Candida parapsilosis* was isolated. In our study, 5 patients revealed Gram-positive coccal infections (3 streptococci, 1 enterococci, and 1 *Staphylococcus aureus*), 2 patients showed Gram-negative bacillary infections (1 *Salmonella* group B and 1 *Klebsiella pneumoniae*), and 1 patient had a Gram-positive coccal infection and a Gram-negative bacillary infection (enterococci + *Escherichia coli*-ESBL).

The clinical symptoms and signs included fever (11 patients, 68.7%), diffuse abdominal pain (6 patients, 37.5%), and left upper quadrant pain or tenderness (6 patients, 37.5%). Physical examination revealed splenomegaly in four patients (25%). Chest radiographs showed left-sided pleural effusions in eight patients (50%). Leukocytosis was noted in 15 patients (93.7%). One patient with AIDS had febrile leucopenia. All patients underwent US and CT. A single abscess was noted in seven patients (43.7%) and multiple abscesses in nine patients (56.3%).

The prognostic factors for splenic abscess are analyzed in Table 2. Antimicrobial therapy was administered in all patients. Fourteen patients (87.5%) recovered under medical treatment. One patient (6.2%) underwent laparoscopic splenectomy, and four patients (25%) underwent percutaneous drainage of their splenic abscess. The mortality rate was 12.5% (2 patients). All four patients who underwent percutaneous drainage and one patient who underwent splenectomy survived, but two (18.2%) of the eleven patients who only had antimicrobial therapy died. The two patients who died separately had the underlying disease AIDS (CD_4_ count: 20 cells/*µ*L, viral load: 21,46,000 copies/mL) and bladder urothelial carcinoma with recurrence and needed to be treated in the intensive care unit.

## 4. Discussion

Splenic abscess is an uncommon entity. The incidence of splenic abscess is estimated to be 0.05–0.7% [1, 2]. The rare occurrence of splenic abscess is evidenced by the study of Altemeier et al. They reported that no splenic abscess was found in reviewing 540 intra-abdominal abscesses [8]. In our study, only 16 patients with splenic abscess were found in the recent 5 years.

Hematogenous spread is the most common cause. The typical examples include patients with septic endocarditis and septicemia. Other risk groups include immunosuppressed individuals (e.g., HIV, malignancy, and diabetes mellitus), trauma, and contiguous spread [5, 9]. In our study, 7 of 16 patients (43.7%) had septicemia including 2 patients with endocarditis. 43.7% of 16 patients had immunodeficiency disorders, such as AIDS, end-stage kidney disease, malignancies, liver cirrhosis, and preterm premature rupture of membranes.

A comparison of epidemiology and symptomatology in patients with splenic abscess in the five periods is reviewed in Table 3. Our patient experienced relatively lower percentage of fever and higher percentage of left lung pleural effusion than those reported by other studies [7, 9–11].

The most common organisms obtained from culture of the abscesses are aerobic microbes, in particular, staphylococci, streptococci, *Salmonella*, and *Escherichia coli* [9, 10]. However, it seems to have geographical variations and population difference. *Klebsiella pneumoniae* was the leading pathogen causing splenic abscess in Taiwan [2]. *Mycobacterium turberculosis* had been reported to be the most common pathogen of liver abscess in Spain [12]. In Thailand, *Burkholderia pseudomallei* had been prescribed to be the most predominant pathogen in a retrospective study of 60 cases with splenic abscess [13]. Regarding fungal splenic abscesses, they were found predominantly in immunocompromised patients [14]. In our study, no specific pathogen was predominant in patients with splenic abscess. It could be limited by fewer case numbers.

As to the treatment of splenic abscesses, intravenous antimicrobial therapy, CT-guided percutaneous aspiration, and splenectomy were the options. An earlier study had shown that the use of intravenous antimicrobial therapy alone resulted in 100% mortality [9]. In recent years, some researches had indicated the success rate of 70.8%–100% in patients with splenic abscess treated with antimicrobial therapy alone (Table 4) [2, 7, 11, 15]. In our study, 68.7% (11/16) of our patients received antimicrobial therapy alone, and 81.8% (9/11) of these patients got recovered. We also found that small and multiple abscesses may respond to intravenous antimicrobial therapy alone even though there was no significant difference (*p*=0.491). The combination of ceftriaxone and metronidazole is the most common empiric antimicrobial treatment in our study.

Percutaneous drainage is an alternative for critically ill patients and for young patients who vigorously attempt to preserve the spleen [16]. Furthermore, percutaneous drainage is only performed when the abscess is unilocular or bilocular with a discrete wall and no internal septa and liquid content [12]. In our study, 4 of 16 patients (25%) underwent percutaneous drainage and all recovered.

The mortality rate varied from 12.4 to 27.6% [7, 9, 11, 15]. Patients with multiple splenic abscesses or immunodeficiency are suggested to have a poor prognosis and high mortality [2, 10]. The overall mortality rate in our study was 12.5% which was consistent with that of previous studies [7, 9]. It is worth mentioning that the two dead patients had serious immunodeficiency and needed to be treated in the intensive care unit (ICU). Because of the limited cases, it is difficult to compare the outcome between the tubercular and pyogenic origin. We speculated that patients with underlying immunodeficiency may also have contributed to these deaths.

## 5. Conclusions

Since there are no guidelines regarding its diagnosis and management, the best therapeutic approach for splenic abscess is still a matter of debate. Based on our experience, patients with splenic abscesses receiving antimicrobial therapy alone were in a relatively high proportion and got a good prognosis especially in patients with small and multiple abscesses. We also noted that mortality may be more related to patients with underlying immunodeficiency. Due to fewer cases collected in our study, further research will be needed to support our study in the future.

## Figures and Tables

**Table 1:** Demographic characteristics, treatment, and outcome for patients with splenic abscess.

Case number	Sex	Age (years)	Predisposing factors	Number of abscesses	Microbiological etiology	Treatment	Outcome
1	F	71	Biliary liver cirrhosis, gallstone s/p cholecystectomy, acute pancreatitis	Single	Alpha-*Streptococcus* species (blood)	AT	Recovered
2	M	75	HTN, infectious endocarditis	Two mixed echoic nodules (2.0 × 2.8 cm and 3.9 × 2.1 cm)	Beta-*Streptococcus* non-ABD (blood)	AT	Recovered
3	M	67	HTN, ESRD, bladder urothelial carcinoma, high grade s/p TURBT, with recurrence s/p robotic radical cystoprostatectomy, acute pancreatitis	Single (4.3 × 1 cm in size)	ESBL-producing *Escherichia coli* (blood), *Enterococcus faecium* (blood)	AT	Died
4	M	32	Chronic pancreatitis with pancreatic duct dilatation and pancreatic stones	Single (2.9 cm)	Not identified	AT	Recovered
5	F	82	Liver cirrhosis, malignant neoplasm of intrahepatic bile ducts, postcholecystectomy	Single (10 × 3 cm)	Beta-*Streptococcus* non-ABD (blood)	PD + AT	Recovered
6	F	51	DM, hyperthyroidism, valvular heart disease, congestive heart failure, infective endocarditis	Single (5.0 × 4.0 cm)	Coagulase (−) *Staphylococcus* (MS-CNS) (blood × 3 sets)	PD + AT	Recovered
7	M	48	AIDS (CD4 count: 59 cells/*µ*L, viral load: 308 copies/mL), anal abscess and anal fistula	Single (4.8 cm wedge)	Not identified	PD + AT	Recovered
8	F	43	Peptic ulcer, duodenitis, chronic hepatitis B	Multiple	*Klebsiella pneumoniae* (liver abscess)	AT	Recovered
9	M	49	AIDS (CD4 count: 20 cells/*µ*L, viral load: 21,46,000 copies/mL)	Multiple	*Mycobacterium avium* complex (culture at bone marrow biopsy)	AT	Died
10	F	77	Chronic liver disease, DM, HTN	Multiple (a 10 cm abscess with smaller abscesses)	*Enterococcus* species (abscess)	LS + AT	Recovered
11	M	48	DM, HTN, pancreatitis	Three (2.4, 1.22, 1 cm)	Not identified	AT	Recovered
12	M	31	DM, alcoholism, chronic hepatitis B, chronic pancreatitis	Multiple	Not identified	AT	Recovered
13	F	81	DM, HTN, ESRD, valvular heart disease	Multiple	Not identified	AT	Recovered
14	F	42	Cholangitis	Three	*Mycobacterium tuberculosis* complex (lung tissue, sputum)	AT	Recovered
15	M	1 d	Prematurity (GA 33 + 6 weeks, BBW 2208 gm), PPROM 10 days s/p complete IAP, necrotizing enterocolitis	Multiple	*Candida parapsilosis* (blood)	Antifungal agents	Recovered
16	F	2	Acute gastroenteritis	Single (11.57 × 8.74 × 12.27 cm)	*Salmonella* group B (abscess, blood)	PD + AT	Recovered

LS: laparoscopic splenectomy; AT: antimicrobial therapy; PD: percutaneous drainage; DM: diabetes mellitus; ESRD: end-stage renal disease; AIDS: acquired immune deficiency syndrome; PPROM: preterm premature rupture of membranes.

**Table 2:** Prognostic factors for splenic abscess.

Variables		Outcome			
	Category (*n*)	Recovered	Mortality	*t*	*p*
		(*n* = 14, %)	(*n* = 2, %)		
Gender	Female (8)	8 (50)	0 (50)	—	0.467^∗^
	Male (8)	6 (75)	2 (25)	—	—
Age (years)	Mean ± SD	48.8 ± 26.8	58.0 ± 12.7	0.467	0.648^∗∗^
Number of abscesses	Solitary (7)	6 (86)	1 (14)	—	1.0^∗^
	Multiple (9)	8 (89)	1 (11)	—	—
Immunodeficiency	Without (4)	4 (100)	0 (0)	—	1.0^∗^
	With (12)	10 (83)	2 (17)	—	—
Underlying diseases	Without (2)	2 (100)	0 (0)	—	1.0^∗^
	With (14)	12 (86)	2 (14)	—	—
Microorganism	GNB (2)	2 (100)	0 (0)	—	0.092^∗^
	GPC (5)	5 (100)	0 (0)	—	—
	GNB + GPC (1)	0 (0)	1 (100)	—	—
	Fungus (1)	1 (100)	0 (0)	—	—
	*Mycobacterium* (2)	1 (50)	1 (50)	—	—
	Sterile (5)	5 (100)	0 (0)	—	—
Treatment modality	AT + LS (1)	1 (100)	0 (0)	—	1.0^∗^
	AT + PD (4)	4 (100)	0 (0)	—	—
	AT alone (11)	9 (82)	2 (18)	—	—
Number of abscesses in patients with AT alone	Solitary (3)	2 (67)	1 (33)	—	0.491^∗^
	Multiple (8)	7 (88)	1 (12)	—	—
Length of stay (days)	Mean ± SD	33.4 ± 24.8	19.0 ± 7.1	−0.797	0.439^∗∗^

GNB: Gram-negative bacillus; GPC: Gram-positive cocci; AT: antimicrobial therapy; PD: percutaneous drainage; LS: laparoscopic splenectomy; ^∗^Fisher's exact test; ^∗∗^*t*-test of independent samples.

**Table 3:** Comparison of epidemiology and symptomatology among five studies.

Variable	Nelken et al. [10]	Ooi and Leong [9]	Chiang et al. [11]	Liu et al. [7]	Present study
Duration of study	1977–1986	1987–1995	1990–2001	2000–2011	2012–2016
Number of cases	189	287	29	28	16
Male : female	125 : 64	163 : 80	18 : 11	13 : 15	8 : 8
Age, mean (years)	Not available	41.4	44	46.5	49.9
Age range	6 mo–82 y	6 mo–92 y	4 y–85 y	4 mo–85 y	1d–52 y
Clinical presentations					
Fever (*n*, %)	131/156 (84)	246/271 (90.8)	26/29 (89.7)	20/28 (71.4)	11/16 (68.7)
Left upper quadrant pain (*n*, %)	61/156 (39)	126/253 (49.8)	9/29 (31.0)	4/28 (14.3)	6/16 (37.5)
Splenomegaly (*n*, %)	62/156 (40)	79/257 (30.7)	5/29 (17.2)	9/28 (32.1)	4/16 (25.0)
Left-sided pleural effusion (*n*, %)	Not available	57/256 (22.3)	3/29 (10.3)	9/28 (32.1)	8/16 (50.0)

**Table 4:** Comparison of recovered and mortality rates with various treatment options in the reviews.

Lead author	Year published	Duration of study	Number of cases	Treatment modality	*N* (%)	Recovered, *n* (%)	Mortality, *n* (%)
Chiang et al. [11]	2003	1990–2001	29	AT alone	24 (82.8)	17 (70.8)	7 (29.2)
				AT + PD	2 (6.9)	2 (100)	0 (0)
				AT + ST	3 (10.3)	2 (66.7)	1 (33.3)
Chang et al. [2]	2006	1986–2004	67	AT alone	20 (29.9)	16 (80)	4 (20)
				AT + PD	21 (31.3)	15 (71)	6 (29)
				AT + ST	26 (38.8)	24 (92)	2 (8)
Lee et al. [15]	2011	1993–2008	18	AT alone	8 (44.4)	8 (100)	0 (0)
				AT + PD	4 (22.2)	2 (50)	2 (50)
				AT + ST	6 (33.3)	5 (83.3)	1 (16.7)
Liu et al. [7]	2014	2000–2011	28	AT alone	18 (64.3)	17 (94.4)	1 (5.6)
				AT + PD	5 (17.9)	3 (60)	2 (40)
				AT + ST	4 (14.3)	4 (100)	0 (0)
				AT + PD + SD	1 (3.5)	0 (0)	1 (100)
Present study	—	2012–2016	16	AT alone	11 (68.7)	9 (81.8)	2 (18.2)
				AT + PD	4 (25)	4 (100)	0 (0)
				AT + ST	1 (6.3)	1 (100)	0 (0)

AT: antimicrobial therapy; PD: percutaneous drainage; ST: splenectomy.
